# The Second European Conference on Zebrafish Genetics and Development

**DOI:** 10.1002/cfg.87

**Published:** 2001-08

**Authors:** Benjamin Feldman

**Affiliations:** The National Institute for Medical Research Division of Developmental Biology Mill Hill London NW7 1AA UK


**Comparative and Functional Genomics**
*Comp Funct Genom* 2001; **2**: 252–256.
**DOI:** 10.1002/cfg.87

**Meeting Review**

### The Second European Conference on Zebrafish Genetics and Development

### University College London, England. April 19–22, 2001

Benjamin Feldman*
*The National Institute for Medical Research, Division of Developmental Biology, Mill Hill, London NW7 1AA, UK*

**Correspondence to:
Benjamin Feldman, The National Institute for Medical Research, Division of Developmental Biology, Mill Hill, London NW7 1AA, UK.
E-mail: bfeldman@nimr.mrc.ac.uk*

Less than five years ago, the zebrafish swam into the limelight as a model genetic organism, with the description of hundreds of mutant phenotypes from screens in Boston and Tübingen (Development 123, 1996). Since that time, much of the zebrafish research community has focussed on identifying the chemically-induced mutations responsible for these phenotypes and on improving zebrafish genomic tools and resources. Other researchers have embarked on new mutant screens, while still others have continued to exploit the amenability of zebrafish embryos to surgical manipulation and microscopic imaging to address questions at the cellular level. Annual conferences on zebrafish development and genetics have provided impressive snapshots of the progress made in these various directions and others, and this year's Second European Conference on Zebrafish Genetics and Development, which took place from April 19–22 at University College of London, was no exception.

### Identification of mutations

A number of speakers announced the identification of mutated genes causing specific phenotypes. In separate talks, **T. Dickmeis (Strasbourg)** and **H. Verkade (San Francisco)** revealed that *casanova* mutants, which lack endoderm and display *cardia bifida*, bear a mutation in a gene related to the SoxF family of transcription factors. **E. Walsh (San Francisco)** showed that the branchial arch defects of *jekyll* mutants and their failure to form an atrioventricular valve in the heart or semicircular canals in the ear result from mutation of an enzyme involved in glycosaminoglycan synthesis, and **S. Horne (San Francisco)** demonstrated that mutation of a protein kinase gene likely to be involved in cell polarity causes the defective morphogenesis of the heart, eye and visceral organs seen in *heart and soul* mutants. Eye defects are also seen in *lakritz* mutants, which lack retinal ganglion cells while displaying an excess of other classes of retinal neurons and glia, and **J. Kay (San Francisco)** showed that this phenotype arises from a mutation in *ath5*, a zebrafish homologue of the transcription factor *atonal*, leading to misallocations of retinal cell fate. In a 'guest fish' talk, **J. Wittbrodt (Heidelberg)** described medaka *eyeless* mutants, which have a failure in eye evagination, and provided evidence that this phenotype results from the disruption of a homeobox gene. **D. Kozlowski (Philadelphia)** showed that *dog-eared* mutants, which do not form cristae in the inner ear, and which have reduced numbers of hair cells and lateral line neuromasts, have a mutation in the *eyes absent-1* gene. **H. Grandel (Heidelberg/Dresden)**

Copyright © 2001 John Wiley & Sons, Ltd.

introduced *no fin*, a new mutant that lacks pectoral fins and cartilaginous gill arches, and presented evidence that *no fin* bears a mutation in a retinoic acid biosynthetic enzyme. Finally, two speakers, **W. Driever (Freiburg)** and **G. Reim (Heidelberg/Dresden)** independently revealed that mutation of a POU-domain transcription factor causes the phenotype seen in *spiel ohne grenzen* mutants, which fail to establish a mid-hindbrain boundary (MHB) and display severe mid- and hindbrain defects. In summary, the cloning of a variety of zebrafish mutations has opened new windows on biochemical pathways responsible for germ layer formation, heart, eye and ear development, regionalization of the brain and other developmental processes.

### New genetic screens

The original screen in Tübingen was very large scale, with 3857 mutagenized genomes screened (2746 F2 families), but this was surpassed by the $\sim$5000 genomes screened in the recently completed 'Tübingen 2000' screen by **Artemis Pharmaceuticals** (co-founded in 1998 by C. Nüsslein-Vollhard and K. Rajewsky) and collaborators. More important than size, perhaps, new screening criteria were employed, and sperm samples from each F1 male were stored for future use. Initial results on one component of this screen, focusing on vasculogenesis and angiogenesis, were presented by **H. Habeck (Tübingen)**. Embryonic vasculature was visualized with alkaline phosphatase and over 700 mutants were obtained, falling into four broad classes: those lacking major vessels, those lacking secondary vessels, those with supernumerary vessels and those displaying abnormal vessel morphology. In addition, **K. Dooley (Boston)** and **C. Klisa (Heidelberg/Dresden)** presented posters on the results of collaborations with Artemis to identify new hematopoeisis mutants and new brain development mutants, respectively. In the hematopoeisis screen, a number of mutants with altered expression of the hemangioblast marker *scl* were obtained, as well as over 100 mutants with decreased numbers of erythrocytes. In the brain development screen, more than 200 mutants were obtained in a variety of phenotypic classes. Four classes were presented, namely mutants with malformations in either the midbrain alone, fore- and midbrain together or in the caudal hindbrain, and mutants with enlarged telencephalons. Results from various smaller-scale screens were also presented. For instance, **R. Dosch (Philadelphia)** described a four generation screen for maternal-effect genes. Because homozygous recessive females are required for this screen, the isolated mutations cannot be zygotically required for female survival or fertility. Despite this limitation, 24 maternal-effect mutants were isolated, displaying defects in either chorion elevation, egg activation, general cell survival, gastrulation movements, axial patterning or primordial germ cell development. **Y. Grinblat (Boston)** introduced results from a clever one-generation screen that combines insertional mutagenesis and haploid screening. Eggs from females mutagenized with an insertional vector were activated with non-functional sperm and grown as haploid embryos. Such embryos were screened for defects in early brain morphology and alterations in neurectoderm marker staining patterns at the neural plate stage, and 19 founder females were identified that transmit defects specific to the forebrain, midbrain or hindbrain. Because the mutagen provides a tag, the cloning of these mutations is potentially straightforward, and is already underway. To conclude, genetic screens continue to generate a variety of interesting new mutants, and the study of these mutants is likely to fuel future research in such areas as vascular, hematopoetic and neural development.

### A zebrafish genome project

The challenge of cloning the growing number of zebrafish mutants will be vastly facilitated by major advances in the state of zebrafish genomics, which have already begun. Most noteworthy is the **Wellcome Trust's** decision to fund the sequencing of the zebrafish genome by the **Sanger Centre**, as announced in October 2000. **J. Rogers (Hinxton)** described the **Sanger Centre's** rationale and sequencing strategy. In addition to its obvious benefit to zebrafish research, comparison of this lower vertebrate sequence with those of mouse and man will likely help to identify evolutionarily conserved regions of non-coding DNA with important functions. Sequencing is already underway and will soon be at full capacity. For this initial phase, a whole-genome shotgun strategy is being used, with 3X coverage of the $\sim$1.7 Gb genome estimated to be completed by December. All primary trace data will be publicly available through the **Sanger Centre's**

Copyright © 2001 John Wiley & Sons, Ltd.

and **NCBI's** servers; indeed, the first 400 000 traces are already available. Because of the mathematical obstacle of assembling millions of non-anchored sequence fragments into contigs, mapped clones will be sequenced in a second phase, estimated to be completed by October 2003. A key resource for this second phase will be the physical map that is being collaboratively assembled by the **Max Planck Institute in Tübingen** and the **Hubrecht laboratory** in Utrecht. **G.-J. Rauch (Tübingen)** described their approach. Clones (averaging 90 to 165 Kb in size) from bacterial artificial chromosome (BAC) libraries are digested to reveal restriction enzyme fingerprints, and commonalties between fingerprints of overlapping clones are identified by analytical software developed at the **Sanger Centre**. Using state-of-the art tools for ongoing high throughput and analysis, well over 100 000 BACs (to achieve $>$10-fold coverage of the genome) will be assembled into minimum tiling paths. At the time of the conference, 25 810 BACs had already been analyzed, 11 447 of which had been assigned to 3254 contigs. A further goal at the **MPI** in Tübingen is to anchor the resulting physical map to a recombinant hybrid panel that is already significantly anchored to a high-resolution meiotic map, thereby allowing the rapid and convenient identification of BAC clones near mapped mutations. Thus, within a few short years zebrafish research will be in its post-genomic phase and the task of positionally cloning mutations will be dramatically eased.

### Reverse genetics in zebrafish

Zebrafish research has been hampered by the inability to directly assess the genetic function of cloned genes, as is routinely done in mice using knock-out technology or in *Drosophila* by P-element insertion. This obstacle is now being removed. In collaboration with **Artemis pharmaceuticals**, members of the **Hubrecht laboratory** have demonstrated the feasibility of pre-screening F1 founder fish by brute-force sequencing to identify carriers of specific mutations. **R. Plasterk (Utrecht)** described this pilot study. In the Artemis screen, tissue samples from each of the F1 founders were routinely stored in addition to sperm. Using primers spanning a large portion of the zebrafish *RAG1* gene, a crucial gene for the establishment of the immune repertoire, DNA isolated from tissue of each of 2687 F1 founders was PCR amplified and sequenced, leading to the identification of carriers of 13 mutant alleles. Three of these alleles had silent mutations, 10 had amino acid changes and one had a premature stop codon. Though daunting in appearance, this approach is surprisingly feasible if an F1 sperm and DNA library is available: reagent costs were under $10 000; the sequencing took one month on a standard ABI sequencer; and fertile carriers can be established from sperm stocks within three months. Luckily, an alternative approach for assessing the function of cloned genes has recently emerged that is much cheaper and much faster, though perhaps less definitive from a genetic standpoint: the use of antisense morpholino oligonucleotides (MOs) to inhibit translation of targeted RNA. One year ago, at the Zebrafish Genetics and Development Meeting at Cold Spring Harbor, **A. Nasevicius and S. Ekker (Minneapolis)** presented a poster on the use of MOs in zebrafish. This year 16 of 56 talks and numerous posters included MO data, testifying to their robust utility in zebrafish. In many cases, MOs were used to confirm an essential role for mutated alleles linked to mutant phenotypes. This was done by recapitulating these phenotypes in wild type embryos injected with MOs targeted to the gene in question. In some cases MOs were used in advance of mutant allele sequencing, in order to first identify which of several candidate genes could be inhibited to produce the mutant phenotype in question. In this way, MOs are facilitating the cloning of mutations by saving time and increasing confidence in the identification of candidate genes. Of course MOs are also being used to investigate the phenotypic consequences of removing genes of unknown function, and certain presentations that included this approach are reviewed in the next section. Thus, reverse genetics has finally arrived in zebrafish. Considering that the sequence of every zebrafish gene will soon be available, the timing couldn't be better.

### New genes and their functions

A number of interesting new genes were introduced in this year's conference, and using MOs it was often possible to complement data on their expression patterns and misexpression phenotypes with the consequences of their inhibition. **C. Houart (London)** built upon her previous identification of

Copyright © 2001 John Wiley & Sons, Ltd. 

forebrain-inducing cells at the anterior neural boundary with her identification of a WNT antagonist that is specifically expressed in these cells and which can confer forebrain-inducing activity to naive cells. MO inhibition of this gene leads to forebrain deficiencies, and reciprocal brain defects were seen when a WNT gene was ectopically expressed, or inhibited with MOs. These studies reveal an essential role for WNT signaling in repressing forebrain development. Also contributing to the understanding of brain patterning, **L. Bally-Cuif (Munich)** described the isolation of *bts1*, a zebrafish gene with homology to *Drosophila buttonhead* . Expression of *bts1* is confined to the MHB after gastrulation. Misexpression of *bts1* can induce ectopic *pax2.1*, which is normally confined to the MHB, and MO inhibition of *bts1* blocks the early expression of *pax2.1*. Therefore, *bts1* is an early regulator of *pax2.1*. Relatively few genes have been described that are specific to ventral ectoderm and the development of epidermis, so **J. Bakkers' (Freiburg)** talk on p63, a p53-related transcriptional repressor involved in ectodermal patterning, was a welcome addition. *p63* is expressed in the epidermal precursor domain (ventral ectoderm) during gastrulation, and its overexpression expands this domain. Consistent with a role in epidermal development, MO inhibition of p63 causes skin lesions in larvae. Intriguingly, *p63* MOs also block pectoral fin bud outgrowth and FGF8 expression in the apical ectodermal ridge.

### Bringing the tools together

One of the great attractions of studying zebrafish is they are nice to look at, and several talks that featured *in vivo* imaging served as a reminder of this. **P. Herbomel (Paris)** presented video recordings documenting movements of early macrophages. These macrophages spread into cephalic mesenchyme, invade adjacent epithelial tissues (brain, retina and epidermis) and then display a wandering behavior as if they are scavenging. In *panther* mutants, which lack a functional macrophage colony stimulating factor (M-CSF) receptor, macrophages fail to invade epithelial tissues, suggesting the possibility that M-CSF serves as a chemoattractant that triggers invasive behavior. **T. Yoshida (Tokyo)** presented particularly elegant studies that employed a tissue-specific promoter to drive tau-eGFP (Green Fluorescent Protein) expression in olfactory sensory neurons of transgenic embryos, and visualized the pathfinding behavior of these neurons, using confocal microscopy. Pathfinding behavior was then compared between embryos carrying transgenes that had been additionally engineered to either enhance or repress protein kinase A (PKA) signaling. In this way, distinct olfactory sensory neuron pathfinding behaviors were correlated to PKA signaling levels, suggesting a native role for PKA signaling in modulating pathfinding behavior. This use of eGFP vectors for high-resolution cellular analysis in the context of transparent zebrafish embryos is particularly exciting, but the generation of transgenic lines of zebrafish remains inefficient. Promisingly, preliminary data from **K. Kawakami (Tokyo)** indicates that inclusion of cis sequences from the medaka Tol2 transposable element in eGFP vectors can significantly enhance their germ-line integration when co-injected with Tol2 transposase. Thus, by combining genetic alterations with high quality imaging, increasingly refined questions are being asked. In a similar way, cellular transplantation can be used to juxtapose cells with distinct genetic conditions. For instance, **N. David (Paris)** demonstrated that expression of Tar* (a constitutively active form of the ALK4 Nodal receptor) in donor cells causes them to join the host endoderm and acquire an endodermal fate. This fate induction/competence restriction does not rely on signals from host endoderm, however, as Tar*-expressing donor cells also differentiate as endoderm in *casanova* mutant hosts, which are devoid of endogenous endoderm. Finally, **Y. Chen (New York)** demonstrated that the zebrafish Nodal protein Squint, but not Cyclops, can behave as a morphogen. First, three genes expressed in response to Nodal signals were shown to respond to ectopic Squint in a graded manner, and secondly, Squint was shown to elicit long distance responses in either Nodal-deficient embryos (*sqt;cyc* double mutants) or Nodal-non-responsive embryos (*Maternal-Zygotic one-eyed pinhead*) harboring a small group of transplanted responsive cells. Thus, it is becoming easier to distinguish possible mechanisms of cellular induction by comparing the responses of wild-type and mutant hosts to grafts or misexpressed constructs.

In conclusion, while the Second European Conference on Zebrafish Genetics and Development will certainly be remembered as the meeting where the zebrafish genome project was inaugurated,

Copyright © 2001 John Wiley & Sons, Ltd.

where MO-based research hit the ground running and where **J. Topczewski's (Nashville)** talk was interrupted by two fire evacuations, it was also memorable for the broad range of excellent science and the increasing sophistication of experimental approaches.

---

The Meeting Reviews of *Comparative and Functional Genomics* aim to present a commentary on the topical issues in genomics studies presented at a conference. These reviews are invited and each represents a personal critical analysis of the current reports and aim at providing implications for future genomics studies.

*The* **Genomics** *Website at Wiley*

**www.wiley.co.uk/genomics**

The **Genomics** website at Wiley is a new and **DYNAMIC** resource for the genomics community, offering **FREE** special feature articles and new information **EACH MONTH**.

Find out more about *Comparative and Functional Genomics*, and how to view all articles published this year **FREE OF CHARGE**!

Visit the **Library** for hot books in Genomics, Bioinformatics, Molecular Genetics and more.

Click on **Primary Research** for information on all our up-to-the minute journals, including: *Genesis*, *Bioessays, Gene Function and Disease*, and the *Journal of Gene Medicine*.

Let the **Genomics** website at Wiley be your **guide** to genomics-related web sites, manufacturers and suppliers, and a calendar of conferences.

WILEY

1516

Copyright © 2001 John Wiley & Sons, Ltd.

